# Dental Literature of the Past and Present

**Published:** 1900-03

**Authors:** 


### Editorial.

### DENTAL LITERATURE OF THE PAST AND PRESENT.

Very few, it is surmised, think of comparing the dental literature of to-day with that of fifty years ago; or, as they receive their journals monthly, stop to consider the enormous strides made during a few years in the development of this side of dental labor. That it has more than kept pace with the progress in other directions, does not require enlargement here.

When a cursory view is taken of the dental periodicals published within the memory of many now active in the dental profession, it is evident that those of less than a half century ago were not even on a level with the standard of thought and practice of that period. With perhaps one exception the few existing were simply advertising sheets with a very limited claim to literary ability, and none at all to original or scientific thought.

It may be said, with some show of truth, that dentistry at that period was too much engaged laying the foundations of a profession to be interested in the solution of literary or scientific problems. While this was then true, it still remains unaccountable that so little was done to build up this better side of professional work in this country. The explanation of this may possibly be found, not in the lack of ability, for that certainly existed, but in the absence of an incentive to work. This came at a later period. The isolation of individuals combined with the feeling that each man must be his own instructor, a feeling that then held supreme mastery of the individual, had much to do with this inaction. Until this was removed, broader thought and more advanced ideas were an impossibility.

It must be evident, as dental history is read, that periodical literature made but slow progress and only grew as associated effort developed. Hence as dental societies became a permanent means of interchanging thought, dental journals remained as they had been, simply as a means to advertise the wares of those responsible for their publication. They contributed little or nothing to the advancement of dentistry, beyond the border-land that exists between trade and profession.

The development of associations was the renaissance of dental literature. With association grew a new development and a demand for better mental pabulum until, at the present time, we find our journals and books worthy the advancement made in other directions. That this change for the better has been mainly due to association, must be conceded. It may be said that the progress noticed would have been accomplished by natural progressive laws, and to some extent this would possibly have been the result; but it is doubtful whether the journals extant to-day could have developed under these existing conditions. The mass of men are rarely moved except by powerful stress, and it was just this want that made those of forty years ago apparently incapable of performing the work that really should have been begun at that period.

When society was added to society, it required an effort of those connected with them to do some original work, and this influence spread. The quiet labor in the laboratory began to be demonstrated on society platforms, and the local, state, and annual gatherings, as these were organized, served to show that here and there a new school of thinkers were being brought to the front. These grew as education advanced in dentistry, and *pari passu*, the character of dental journalism began to assume an altogether different character. Certain portions of it, at present, compare very favorably with the literature of the older professions, indeed, in some respects, much of the reading matter is in advance in scientific ability. That this will continue proportionately to progress as dental education takes higher ground, needs no argument.

There is still, however, left an unsatisfactory remnant of the old condition. The advertising sheet is yet with us made up of abridged restatements from other journals, which, while they may do some good, exist only by crucifying the original work of writers. It is probably true that these are prone to prolixity, but it is not equally true that editors of trade journals are always the best judges of what a writer should say, or leave unsaid. A recent editorial in one of these sought to excuse this mutilation by assuming that writers used too many words, and, by indirection, intimated it was for their good and that of the readers that the articles were cut down and thus became better adapted to the comprehension of those who read the journal. While it is true that there is a tendency to prolixity, it is assumed that an author is the best judge of what he meant to say and how he should say it. At all events it is not exactly honorable to steal the grain and throw away the so-called straw, for it must be remembered that both are essential in giving life.

While dental periodical literature of this character will always be with us, there is a bright side even here; and this is evidenced by the fact that the supply houses have found it necessary to meet advancing conditions by placing men of intellectual force in control, and this gives a prospect for a better state of things in the future.

The vast difference between now and then, or the present and past, must be apparent in a survey of the contents of the few journals that have risen to a higher level. Instead of a few pages of reading matter, these have expanded from sixty to ninety pages monthly. In place of dull text, this is enlivened by the highest skill of the engraver's art. Every effort is made to give variety in addition to the best thoughts. The criticism has been made that those journals that present the best work do not receive this at first hands, but secure it from various societies. Such a criticism has no force, for this must continue to be the only channel through which original workers seek to give publicity to their labor. An individual who may have spent months in laboratory investigation, or years in clinical observation, naturally desires to meet his colleagues and have their opinion of his work, and this is not only natural, but it is important, for the criticism received furnishes a standard for judgment. Then the paper goes to that larger audience, the readers of the selected periodical, and receives a wider consideration. In that journal, care is taken that the writer shall not be abridged by a single sentence. Its fate thereafter is left to the conscience of those who republish.

It is useless, perhaps, to speculate as to the number of readers of our present literature. There is no question but the number who carefully read and digest the matter contained in journals, is growing rapidly, and that the large army of dental operators who only read the advertisements are happily growing smaller. The editor who, by his occupation, is forced to read over and over again the matter in his own journal, feels equally constrained to read everything original in his exchanges, and by this continued contact with the best thought becomes, necessarily, better able to judge of the value of that thought and its probable effect on his profession. The reader who fails to follow the paper with the discussion, comprehends only in part the real value of the essay; in fact it frequently occurs that in these discussions matter is brought out worthy a more permanent place in our literature, but, owing to its obscure position, it lies buried beyond hope of resurrection. The close reader of the INTERNATIONAL DENTAL JOURNAL, with its eight hundred and more pages yearly, must have been impressed with this fact. It is much to be regretted that so much of value is practically lost. This applies equally to other journals, organs of society work. The fact is apparent, but the remedy is not by any means clear. Something might be done by those giving valuable ideas through "Casual Communications" or "Incidents of Practice," if they would enlarge these into brief papers for publication. The dental world cannot afford to part with a single original idea.

The growth of dental literature of a high order has been more pronounced abroad than in this country. The dental periodicals of Europe are superior to most of those published here, in that they devote their pages to truly scientific work, and do not permit in them any matter that detracts from the dignity of a profession. The English, French, and German journals teem with valuable scientific articles, and the same may, with equal force, be said of other countries and other languages. These, unfortunately, do not always get into English dress. The journals have associated with them, directly or indirectly, the best scientific minds in the dental profession in the several countries, with the result that they are conducted with a dignity commensurate with the work to be accomplished. The "funny man" has no place there and should have none here. As an evidence of the thoroughness in treating subjects, the October number of the *Osterreichisch-ungarische Vierteljahrsschrift für Zahnheilkunde* (*Austria-Hungary Quarterly Journal of Dentistry*) has an article upon "The So-called Early Extraction of the Sixth-year Molars," and this somewhat over-written theme is illustrated by ninety-seven cuts. This is only one of a number of more or less valuable articles on apparently simple subjects, but all treated in a thoroughly scientific manner. Of these is one by Röse, of Munich, on "Mouth Washes," which contains some original investigations worthy of consideration hereafter.

It is only by a broad examination of the journalism of the dental world that we can arrive at any just comprehension of the progress made in a comparatively few years in dental periodical literature, and he who confines his scope of observation to his own country secures but a contracted idea of its progress and of its value. We must get beyond our insular exclusiveness and egotistic assumption that, unfortunately, leads some minds to believe that there is nothing outside of America that possesses any real value, not only in dentistry, but in other things. If these prejudiced individuals would open their minds to the work that is being done in other fields of dental labor, they might be able to discover that the highest conception of dental journalism is not confined to this country, nor has it been reached here except by two or three periodicals. It is hopeless to expect any marked improvement in dental journals, while a large body of men in our ranks are satisfied with the reading of abstracts and digests. If it be not a sign of partial culture, it is at least an evidence that the dental profession in America, taken as a whole, has not arrived at an intellectual standard enabling them to appreciate their own literature, and until that grade of cultivation be reached, it cannot be expected that the dental literature of the world will receive much consideration.

